# Differential neural encoding of sensorimotor and visual body representations

**DOI:** 10.1038/srep37259

**Published:** 2016-11-24

**Authors:** David Perruchoud, Lars Michels, Marco Piccirelli, Roger Gassert, Silvio Ionta

**Affiliations:** 1 The Laboratory for Investigative Neurophysiology (The LINE), Department of Radiology and Department of Clinical Neurosciences, University Hospital Center (CHUV) and University of Lausanne (UNIL), Lausanne, Switzerland; 2 Institute of Neuroradiology, University Hospital Zurich, Zurich, Switzerland; 3 Rehabilitation Engineering Laboratory, Department of Health Sciences and Technology, ETH Zürich, Zurich, Switzerland

## Abstract

Sensorimotor processing specifically impacts mental body representations. In particular, deteriorated somatosensory input (as after complete spinal cord injury) increases the relative weight of visual aspects of body parts’ representations, leading to aberrancies in how images of body parts are mentally manipulated (e.g. mental rotation). This suggests that a sensorimotor or visual reference frame, respectively, can be relatively dominant in local (hands) versus global (full-body) bodily representations. On this basis, we hypothesized that the recruitment of a specific reference frame could be reflected in the activation of sensorimotor versus visual brain networks. To this aim, we directly compared the brain activity associated with mental rotation of hands versus full-bodies. Mental rotation of hands recruited more strongly the supplementary motor area, premotor cortex, and secondary somatosensory cortex. Conversely, mental rotation of full-bodies determined stronger activity in temporo-occipital regions, including the functionally-localized extrastriate body area. These results support that (1) sensorimotor and visual frames of reference are used to represent the body, (2) two distinct brain networks encode local or global bodily representations, and (3) the extrastriate body area is a multimodal region involved in body processing both at the perceptual and representational level.

Human beings can effortlessly recall perceptions even in absence of the appropriate sensory information^1^, e.g. when we mentally evoke a familiar landscape. Commonly defined as “mental imagery”, such a mental reproduction of physical objects’ properties is not limited to perception, but also it extends to movements^2^. In this case it is defined “motor imagery” and is characterized by the activation of sensorimotor representations even in absence of overt execution^3^. Since the dawn of experimental psychology, a particular interest in motor imagery has driven the attention of the precursors of today’s cognitive neuroscience. Alexander Bain’s “Simulation Theory”^4^ proposed that motor imagery and overt movements rely on similar cognitive mechanisms and neural underpinnings. More recent evidence supports this view, showing that the time to imagine and perform a specific action is proportional^5^, and that imagined and executed movements activate partially overlapping brain networks^6^.

An objective measurement of the temporal properties of motor imagery is provided by a cognitive task called “mental rotation”, in which participants judge the laterality of rotated images while response times (RTs) and accuracy are recorded^7^. In case of bodily images, mental rotation depends on the nature of the images. When hand images are mentally rotated, the performance strongly depends on image orientation (RTs linearly increase from 0° to 180° rotation and vice versa until 360°)^8^ and is influenced by actual proprioceptive input^9^. Conversely, mental rotation of full-bodies is less dependent on orientation^10^ and proprioception^11^. In addition, the absence of proprioceptive input (as after complete spinal cord injury) affects the interplay between visual and sensorimotor components in the representation of the disconnected body parts (feet, in the case of complete spinal cord injury)^12^. Thus, previous work suggests that mental rotation of local bodily images (hands) recruits mainly sensorimotor mechanisms, while mental rotation of global bodily images (full-bodies) is mostly based on visual mechanisms. On this basis, it can be hypothesized that, at the neural level, mental rotation of hands would be associated with the activation of prefrontal, pre-central, and post-central regions, while mental rotation of full-bodies would recruit more strongly the temporo-occipital cortex. Only very few studies compared mental rotation of body parts and full-bodies, only behavioral data were recorded, and the two classes of used images differed in terms of visual aspects of the target item^13^,^14^. To provide relevant information about the neural substrates encoding local (hands) *versus* global (full-bodies) bodily representations, we studied the behavioral and neuroimaging counterparts of mental rotation of hands and full-bodies, in a within-subject fashion, and with comparable visual stimuli.

Different studies investigated the neural underpinnings of mental rotation of either hands or full-bodies, separately. Mental rotation of hands has been associated with activity in prefrontal, precentral, postcentral, and parietal regions^15^,^16^,^17^. Conversely, mental rotation of full-bodies activated mainly temporo-parieto-occipital regions^18^, including the so-called extrastriate body area (EBA) located in the middle-inferior temporo-occipital gyrus^19^. This evidence suggests that distinct neural networks are specialized to process local *versus* global body representations, in that local representations are based on a sensorimotor frame of reference while global representations on a visual one. However, these assumptions must be considered with caution, as none of the previous studies performed a direct and within-subject comparison between the brain activity associated with mental rotation of hands versus full-bodies. Such a direct comparison might reveal the neural counterparts of this behaviorally-based theory. To better clarify the neuro-cognitive mechanisms associated with local and global aspects of body representation, in a within-subject fashion, we recorded brain activity while healthy participants judged the laterality of pictures of hands and full-bodies presented in different orientations from upright (which implies mental rotation). To exclude potential biases due to visual aspects of the images, we extracted the hand image from the full-body one. Thus, the target images (hands for both hand and full-body stimuli) were perfectly comparable in terms of gender, age, race, posture, etc. (Fig. 1A). This approach allowed us to provide objective measurements of both the behavioral and neural responses associated with mental processing of local *versus* global body representations, with eminent insights onto the interplay between sensorimotor and visual frames of reference involved in body representation.

## Results

### Behavior

In accordance with previous studies^11^, RTs were analyzed by means of a 3-way repeated measures ANOVA with stimulus (hands, full-bodies), laterality (left, right), and orientation (0°, 90°, 180°, and 270°) as main factors. The 2-way interaction between stimulus and orientation was significant [F(3,45) = 11.8; p < 0.05]. For both hands and full-bodies, the RTs for stimuli presented at 180° were longer with respect to all the other orientations (all p < 0.05). Interestingly, the Bonferroni corrected post-hoc comparisons showed that, for images presented at 0°, participants were faster in mentally rotating hands (1037 ms) than full-bodies (1180 ms, p < 0.05). Conversely, for images presented at 180°, participants were significantly faster (p < 0.05) with full-bodies (1350 ms) than hands (1519 ms) (Fig. 2). Thus, in line with previous evidence, the mental rotation function (non-monotonical increase of RTs as a function of stimulus orientation) was more pronounced for hands than full-bodies^11^. Further behavioral effects generally confirmed previous work and are reported in detail as Supplementary Material (results).

### Brain Activity

**Figure 1:**
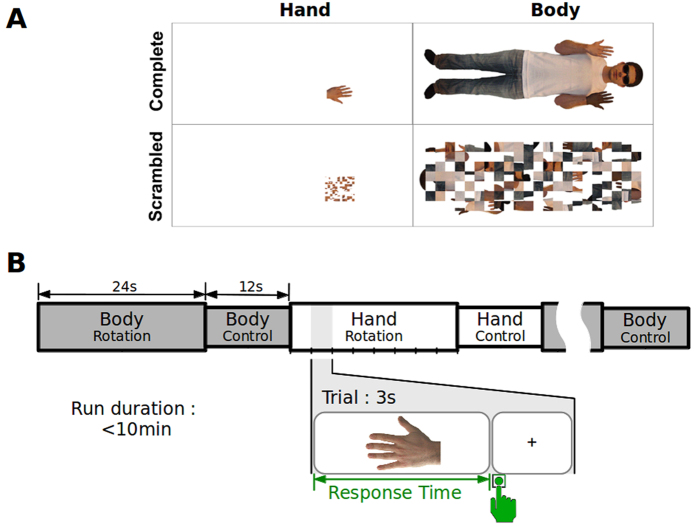
Stimuli and Procedure. **(A)** Experimental Images. Each image was presented twice, either complete or scrambled. **(B)** Schematic representation of an experimental run. During the “rotation” blocks, complete images were presented and participants performed mental rotation. During the “control” block, scrambled images were presented and merely observed by the participants. The bottom line shows an example of mental rotation trial.

**Figure 2:**
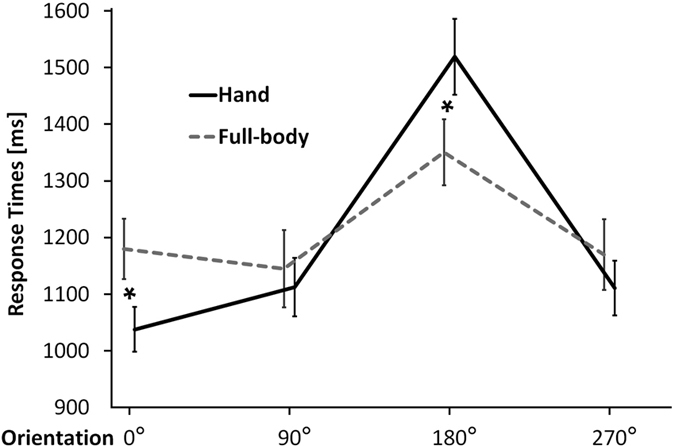
Stimulus-dependent modulation of the mental rotation function. Mean response times (RTs) as a function of stimulus and orientation. With respect to mental rotation of full-bodies, mental rotation of hands is more strongly affected by the orientation of the images. Error bars represent standard errors. Asterisks represent significant differences between hands and full-bodies (p < 0.05).

**Figure 3:**
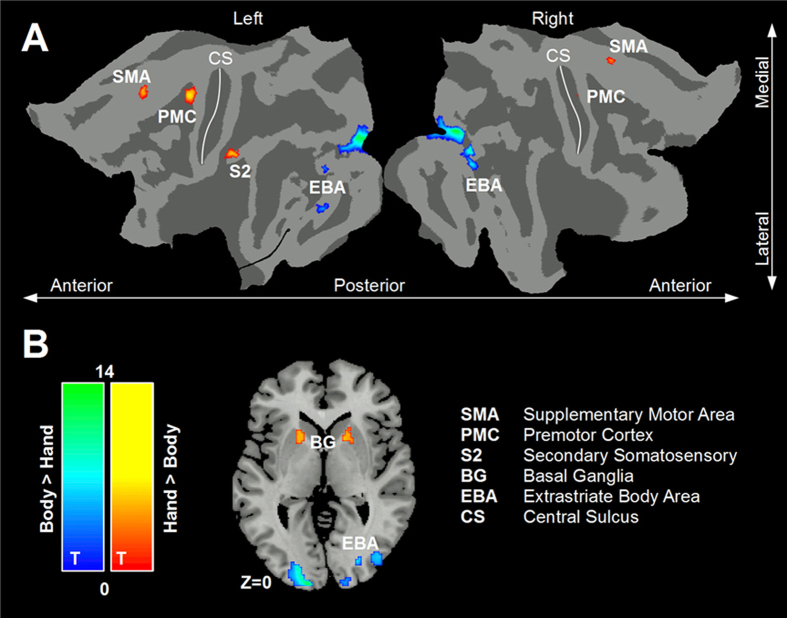
Local vs. global body representations. Direct comparison between the brain activity elicited by mental rotation of hands versus full-bodies. Mental rotation of hands (local body representations) activated the sensorimotor network (red-to-yellow). Mental rotation of full-bodies was associated with stronger activity within the visual network, including the extrastriate body area (blue-to-green). Activation clusters included both (**A**) cortical regions, represented on a flattened brain surface, and (**B**) subcortical regions represented on an axial brain slice.

All the activated clusters and related statistics are reported in Table 1 and graphically represented in Fig. 3. To dissociate the brain activity associated with visual perception from mental representation of the experimental images, in two control conditions participants observed the scrambled versions of the hand and the full-body images, respectively (Fig. 1A). On this basis, the contrast hands > full-bodies showed the regions predominantly activated during mental rotation of hands with respect to mental rotation of full-bodies (t > 4.26; p < 0.05; FDR corrected). These regions were part of the sensorimotor network and comprised, bilaterally, the supplementary motor area (SMA), premotor cortex (PMC), and basal ganglia (BG), plus the left secondary somatosensory cortex (SII). In the left hemisphere, the left-SMA cluster (250 mm^3^) was located in the medial frontal lobe (95% of the voxels) and comprised BA 8 (83% of the voxels). The left-PMC (923 mm^3^) cluster was located in the precentral gyrus (93%) and comprised BA 6 (99%). The left-BG cluster (583 mm^3^) was located in the left putamen (72%), caudate nucleus (18%), internal capsula (7%) and pallidum (1%). The left-SII cluster comprised two sub-clusters. The first SII sub-cluster (564 mm^3^) was located in the supramarginal gyrus (79%) and inferior parietal lobule (21%) and comprised BA 2 (65%) and BA 48 (28%). The second SII sub-cluster (105 mm^3^) was located in the inferior parietal lobule (83%) and the postcentral gyrus (16%) and comprised BA 2 and BA 3 (49% and 48%). In the right hemisphere, the right-SMA cluster (56 mm^3^) was located in the medial frontal lobe (100%, BA 6). The right-PMC cluster (105 mm^3^) was located in the precentral gyrus (100%, BA 6). The right-BG cluster (423 mm^3^) was located in the putamen (47%), caudate (24%), internal capsula (21%), and pallidum (5%).

**Figure 4:**
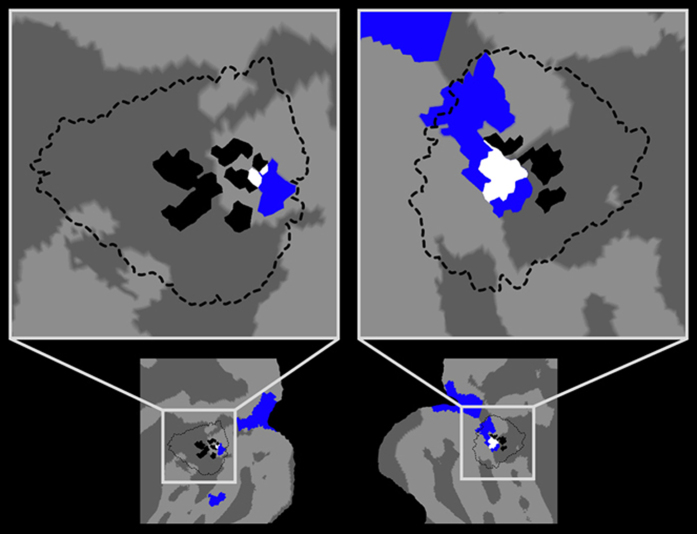
EBA localization. Overlap (white) between the brain activity during mental rotation of full-bodies within the functionally localized EBA (blue) and the peak activity voxels reported in 30 previous studies (black) (Supplementary Table S1). Both the overlap and the activation due to mental rotation of full-bodies were comprised within the regions (dashed line) typically described as EBA (Supplementary Table S1).

The opposite contrast, full-bodies > hands, showed the brain areas more strongly activated during mental rotation of full-bodies (with respect to hands), namely the visual network. These clusters were located within the regions identified by the EBA-functional localizer (t > 4; p < 0.05; FDR corrected), and included the middle occipital gyrus (MOG), fusiform gyrus (FG), and EBA in the left hemisphere, as well as the MOG in the right hemisphere. The left-MOG cluster (1977 mm^3^) included the MOG (70%), the inferior occipital gyrus (11%), the calcarine cortex (8%), and the superior occipital gyrus (4%). This cluster comprised BA 18 (60%) and BA 17 (36%). The left-FG cluster (355 mm^3^) covered the FG (81%) and the inferior temporal gyrus (18%). This cluster was almost entirely located in BA 37 (99%). The left-EBA cluster (54 mm^3^), covered the inferior occipital gyrus (58%) and the MOG (26%), and comprised BA19 (81%). The right-MOG cluster (5117 mm3) covered the MOG (38%), inferior occipital gyrus (20%), inferior temporal gyrus (13%), superior occipital gyrus (9%), cuneus (5%), and FG (4%). This cluster comprised BA 18 (35%), BA 19 (34%), BA 17 (15%), and BA 37 (11%). Thus, mental rotation of full-bodies bilaterally activated parts of the inferior occipital gyrus corresponding to the functionally defined EBA (Fig. 4).

## Discussion

Human beings automatically create local and global mental representations of the body, e.g. to adequately interact with the environment. The integrity of such bodily representations is not obvious and requires the proper functioning of specific neural mechanisms. Here, we report neuro-behavioral data showing that two distinct patterns of cortical activity are selectively associated with mental representation of local body-parts *versus* global whole-body. We assessed the characteristics of local and global body representations by implicitly asking participants to mentally rotate images of hands and full-bodies, respectively. The behavioral data showed that the profile of mental rotation of hands (distribution of RTs as a function of image orientation) was more strongly influenced by anatomically relevant constraints, with respect to full-bodies. At the neural level, the direct and two-way comparison between mental rotation of hands and full-bodies indicated the stronger activation of a set of sensorimotor regions during mental rotation of hands (with respect to full-bodies), and the stronger activation of a set of visual regions during mental rotation of full-bodies (with respect to hands). Only two studies investigated the differences between mental rotation of body parts and full-bodies in healthy^13^ or clinical populations^14^. These studies recorded only behavioral data and used different classes of images for the two categories. Thus, to our knowledge the present study is the first to directly compare, in a within-subject fashion, and with comparable target stimuli, the behavioral and neuroimaging data related to local (hands) *versus* global (full-bodies) body representations. The present data support that local body representations are more strongly based on sensorimotor processing, possibly extending to movement simulation mechanisms^20^. Conversely, global body representations are more robustly based on visual processing, possibly involving mainly components of visuo-spatial reasoning^18^. Unraveling the neuro-behavioral basis of the distinction between local (sensorimotor) and global (visual) bodily representations, we provide a detailed view of where different aspects of body representation are distinctly processed in the human brain. The present findings can have important clinical benefits, offering the baseline reference framework and the experimental approach to assess, monitor, and restore possibly distorted body representations as a consequence of e.g. neural injury and/or degeneration^21^,^22^.

### Mental rotation of hands and the sensorimotor network

The present behavioral data show that mental rotation of hands was strongly affected by image orientation, leading to a non-monotonic increase of the RTs profile at 180°^23^. Such a dependency on orientation is considered a sign of the recruitment of sensorimotor mechanisms^24^, i.e. the simulation of the movement through which one could place one’s own body part in the position of the image^25^. On this basis, our behavioral data confirm the key role of sensorimotor mechanisms in hand-centered mental representations^26^. This interpretation is further supported by our neuroimaging data. Indeed, we found that the sensorimotor network increased its activity during mental rotation of hands, including SMA, PMC, SII, and BG. These regions have been consistently associated with mental rotation of hands. While the most common view links mental rotation of hands to activity in SMA and PMC^16^,^27^,^28^, different studies highlighted the importance of other regions of the sensorimotor network, including SII^15^, and BG^29^. Linking regions previously reported in isolation, our results further extend this evidence by showing that these regions work in parallel, as parts of a widespread network. These findings indicate that local body representations are encoded by the activity of not only specific regions, as previously shown, but also by a widespread brain network including both cortical and subcortical structures and selectively centered on the sensorimotor system.

Why should such a large network be necessary for a relatively simple task such as mental rotation of body parts? It is worth noting that many different aspects of body representation are simultaneously processed during mental rotation. Based on the Simulation Theory, during mental rotation of body parts we use our own body as a reference and we simulate the movement that would bring our body part into the position of the displayed body part. To this aim, we have to know where our body part is located with respect to the body and, hence, have to plan the necessary movement. Thus, at least two aspects are involved in this sensorimotor simulation: one (somatosensory) aspect is more focused on retrieving information on the actual state of the body part; another (motor) aspect is more focused on the transition from the actual status (our own body part) to the target status (as the displayed body part). In this vein, we interpret the activation of the sensorimotor network as the result of both somatosensory and motor components of mental rotation of hands. In particular, for the somatosensory component, we found activity in SII. This region plays an important role in encoding the position of a body part with respect to the whole body both in humans^15^ and non-human primates^30^ and it has been involved in movement execution, observation, and simulation^31^. Accordingly, we interpret the activation of SII as the neural counterpart of encoding the actual (somatosensory) state of the hand with respect to the body, to be further used as reference by the motor component. Indeed, during mental simulation of movements, SII increases its functional connectivity with SMA^32^ and we found activation of SMA and PMC. This latter possibly encodes the motor aspect of mental rotation. This is in line with previous studies which used similar experimental protocols and observed the activation of SMA^28^ and PMC^33^. During mental rotation of hands we also found the activation of BG. It is worth noting that BG project to prefrontal regions^34^, are consistently reported by studies on motor imagery^35^, and their lesion is associated with impaired performance in motor imagery tasks^36^. On this basis, we interpret the activation of SMA, PMC, and BG as the neural counterpart of the motor component of mental rotation. Considering that mental rotation of hands is just one of the tasks used to activate local body representations, we propose that local body representations comprise at least these two components (somatosensory and motor) and that there is a continuous and mutual exchange of information between them^37^. Due to the intrinsic limitations of fMRI and the experimental protocol, we could not disentangle temporal features of the recorded brain activity (which regions are activated first). Further studies are required to clarify this important point, using an investigation technique, such as EEG, specifically designed to understand temporal dynamics of cognitive processes.

### Mental rotation of full-bodies and the visual network

Consistently with previous studies^13^, here we found that mental rotation of full-bodies was weakly affected by the orientation of the image. Considering the stronger impact of orientation on mental rotation of hands (despite the fact that the target hand was exactly the same in both stimuli), these data could be the result of the adoption of two different strategies: “effector-based” versus “perspective” mental transformations, respectively. While effector-based transformations use an egocentric frame of information processing, perspective transformations use an allocentric frame of reference and show higher degrees of physical flexibility^26^, are less influenced by biomechanical constraints^38^, and are less affected by anatomical plausibility^39^. Along this line, if a stereotyped “head” is drawn on the abstract objects typically used in mental rotation studies^41^, the impact of stimulus orientation decreases^40^. Thus, mental rotation of hands should trigger an effector-based transformation, which could result in the activation of mostly somatosensory and kinesthetic representations. Conversely, the full-bodies should elicit perspective transformations and predominantly activate visuo-spatial representations^42^. In support of this view, the present EBA activation shows that mental rotation of full-bodies selectively activates visuo-spatial representations of the body encoded by higher regions of the visual brain network.

Typically, EBA is functionally defined, thus its exact location varies across different studies and different designs^43^. The location of the present EBA-clusters reasonably overlapped with the stereotaxic location of EBA in previous studies^44^. In particular, the coordinates of the present EBA clusters’ centroid were within the range defined by 30 previous studies (Table S1). On this basis, we propose that we identified EBA and that this region is part of the neural substrate implied in processing mental whole-body representations. Interestingly, in a region anatomically overlapping with our EBA, the pattern of activity is parametrically modulated as a function of the speed of mental rotation of bodies^45^. However, any conclusion about EBA based on the results of this last study^45^ has to be considered with caution, due to the absence of an EBA-specific functional localizer. Using a EBA-specific functional localizer, we found that mental rotation of bodies (but not hands) recruited EBA. Previous studies showed that EBA, on the one hand, contributes to the creation of the sense of owning and being situated within a body^46^,^47^. On the other hand, it is involved in allocentric body representations^48^, regardless of the identity of the visualized body (oneself or another person)^49^. Thus, EBA could be responsible for the generation of body ownership in allocentric perspectives, independent from body identity. On this basis, we propose that the stronger activation of EBA during mental rotation of bodies could be a sign that participants embodied the full-body image presented in allocentric perspective. This view extends the functions of EBA to the representational level for body ownership during allocentric perspective taking.

What is the exact role of EBA at the representational level? A previous study fostered the idea that EBA is involved in creating detail-based body representations (body parts)^50^. Conversely, our data show that EBA is activated by whole-body stimuli, and not body parts. This apparent inconsistency might be due to differences in experimental designs, scanning procedures, or data analyses. For instance, while in the study by Costantini *et al*.^50^, the stimuli comprised only body parts, here we directly compared mental rotation of body parts (hand) and full-bodies. In addition, Costantini *et al*.^50^ used explicit imagery (participants were asked to consciously create visual images of specific body parts), while here we used implicit imagery (the laterality judgment implies mental rotation). For these reasons, on the one hand, our data support the conclusion of Costantini *et al*.^50^ that EBA is involved at the representational level of body processing. On the other hand, as our task was focused on the hands also for the full-body stimuli, we further suggest that EBA plays a key role in merging piecemeal local body representations (hands) into a more complete global body representation (full-body), to be further processed by later occipito-temporal regions^51^. In this vein, the present study extends the possible functions of EBA, suggesting that it is a crucial neural hub to encode multimodal body-specific information in the process of creating (from local to global) whole-body representation.

## Conclusions

The results of this study illustrate how our brain is specialized in representing visual and sensorimotor aspects of our body. Merging brain imaging and experimental psychology, we showed selective changes in the cortical patterns of activity associated with local and global body representations. These two fundamental aspects of body representation were neurally encoded by the activity of two distinct networks, i.e. sensorimotor regions for local components (hands) and the visual regions for global components (body). Importantly, the target feature of both the hand- and body-images used here, was exactly the same (the same hand was presented in isolation or attached the appropriate body). Thus, it can be concluded that the activation of the sensorimotor or visual networks was due to contextual contingencies (presence or absence of the body around the hand). The activation of the sensorimotor network during mental rotation of hands suggests the involvement of movement simulation mechanisms. The involvement of the visual network during mental rotation of bodies suggests that visuo-spatial reasoning plays a central role in this task. Accordingly, we showed that EBA, a region classically (but not exclusively) associated with visual perception of human bodies was actively involved also in the mental representation of the body. Previous studies showed that EBA is involved in a broad set of body-related functions, including visual^52^ and tactile^53^ perception of human bodies, execution^54^ and imagination of body movements^55^, and integration of multisensory bodily information^47^. The present results are in line this evidence and extend the role of EBA to the representational level, de facto including the construction of a full-body representation in the body-related functions already attributed to EBA (e.g. motor control, multisensory integration, and body ownership). Altogether, this study provides insights for future investigation of the plastic interplay between sensorimotor and visual representations of the body as a function of individual and contextual factors, in healthy and clinical populations.

## Methods

### Participants

Sixteen right-handed healthy participants (7 female, 24.7 ± 3.8 y.o.) took part in the experiment. All participants had normal vision and signed a written informed consent prior to the experiment. The protocol was approved by the local Ethics Committee of the University of Zurich and the experiment was conducted in accordance with the Declaration of Helsinki 1964.

### Procedure and Stimuli

The fMRI session consisted of three runs, two of which comprised blocks of mental rotation. The third run was used as a functional localizer to identify EBA in each participant. In the mental rotation runs, participants observed images of hands or full-bodies. Images were displayed in the center of the computer screen (12° × 12° of visual angle) and a projector. The projector displayed the images onto a translucent screen positioned in the MRI scanner, behind the participants’ head. We used a custom-made back-projection mirror box positioned in front of the participant’s eyes, inside the magnet, to visualize the images projected on the translucent screen. For the hand images, participants were instructed to judge as quickly and accurately as possible the laterality of the hand. The full-body images represented an avatar person with one darkened hand. In this case, participants judged which hand was darkened^11^. For both types of images the target hand was exactly the same (size, orientation, view, gender, age, race, etc). RT was recorded for each image and was defined as the delay between the image onset on the screen and the participant’s response. Each image was presented in four different orientations (0°, 90°, 180° or 270°). All orientations were considered as clockwise rotations from the canonical upright (fingers pointing upright). The scrambled images were created by splitting the original image (hand or body) in a fixed number of squares and randomizing the position of these squares. The number of squares was the same for the hand and the body images. During the mental rotation task, the images remained visible on the screen until a response was given, with a maximum duration corresponding to one fMRI volume (3 sec). After the participant’s response, the image was replaced by a fixation cross until the following trial started (Fig. 1B). Before the fMRI session, participants familiarized with the task outside the scanner. In order to avoid priming effects, the images presented during the training phase were rotated in different angles (45°, 135°, 225°, 315°) with respect to the experimental ones.

To provide responses, participants held two MRI-compatible pear-shaped pneumatic buttons, one in each hand, and clenched the left or right hand to indicate a left- or right-lateralized image. To control the motor component of this response, in a third control condition participants executed a simple stimulus-response task according to a pre-defined color coding (left or right hand clench in response to red or green square, respectively). This control condition helped us to control for any additional activation due to the hands’ movement. Prior to the experiment all participants were enrolled in a training phase, including both the mental rotation and stimulus-response trials. Only after the successful completion of the training, participants were admitted to the fMRI phase. In this way we made sure that all participants were able to perform all the parts of the experiment and were not color-blind.

Each experimental run was composed of 16 blocks, each containing only one image type (hand or body). Each block comprised eight trials with all the possible combinations (left/right, and four orientations), lasting 24 s, and was followed by the presentation of the corresponding scrambled image for 12 s. The EBA functional localizer consisted in blocks of observation of bodily images interleaved with blocks of observation of an abstract shape. It was composed of 3 blocks, each containing 7 images interleaved with a blank screen, and lasting 21 seconds. After the recording of the functional brain images, a structural brain image was collected for each participant.

### fMRI Data Acquisition

Functional and structural MRI scans were collected using a 3 T SIEMENS MAGNETOM Skyra scanner, operating at the University Hospital of Zurich (USZ). Each functional scan run of mental rotation comprised 200 scans, the EBA functional localizer comprised 85 scans. The following acquisition parameters were used for all functional scans: voxel size 3.0 × 3.0 × 3.5 mm, 28 interleaved slices (whole-brain coverage), TR 3 s, TE 30 ms, matrix size of 72 × 72 voxels. The T1-weighted anatomical images (0.9 mm isotropic voxels, 192 sagittal slices, TR 1.9 s, TE 4.9 ms) were collected using a magnetization-prepared rapid acquisition gradient echo sequence (MPRAGE).

### fMRI Data Analysis

fMRI data were analyzed with SPM8 (Wellcome Department of Cognitive Neurology, Institute of Neurology, UCL, London, UK). In accordance with standard procedures, the data preprocessing included motion correction (all participants <1.5 mm), slice-timing, coregistration, normalization, and smoothing (Supplementary Material). The time series of functional images obtained for each participant were analyzed separately. The effects of the experimental paradigm were estimated on a voxel-by-voxel basis using a general linear model^56^. Each experimental block was modeled using a boxcar, convolved with a canonical hemodynamic response function chosen to represent the relationship between neuronal activation and blood flow changes. Single-subject models were used to compute two contrast images for each participant, representing the estimated amplitude of the hemodynamic response for the hand and full-body conditions relative to the respective control conditions (scrambled images). The scrambled images of both hands and full-bodies were respectively modeled as two regressors defined as boxcar functions convolved with the hemodynamic response and therefore constituted the baseline contrasts for each corresponding experimental image. The resulting contrast images were subsequently analyzed using a random effect approach.

Cognitive tasks similar to the one used in the present study, activated the sensorimotor circuit, including the prefrontal^27^, premotor^57^, and secondary somatosensory cortex^15^, as well as the BG^35^. On this basis, we ran a second-level region-of-interests (ROI) analysis including both functional (the EBA functional localizer) and anatomical regions (somatosensory, motor, premotor, and prefrontal cortices, plus the BG). For the functional ROI (the EBA localizer), the fMRI data were analyzed according to the task factor (observation of body *versus* abstract shape. The resulting group-level activation map was statistically corrected for False Discovery Rate (FDR) (*p* < 0.01; t = 6.11)^58^ with a minimum cluster size of 10 voxels (k > 10)^59^. The anatomical ROI was created using the WFU atlas toolbox^60^ implemented in SPM, and included the motor and somatosensory regions [Brodmann Areas (BA) 1, 2, 3, 4 and 6] as well as the BG. Both the functional and the anatomical ROIs were combined in a single ROI, on which the second-level analysis was performed. At the second level the two contrast images for each participant (hands > scrambled hands; full-bodies > scrambled full-bodies) were entered into a paired t-test contrast analysis with 16 pairs. Thus, for each of these two contrast images, we excluded the influence of visual processing on the resulting brain maps related to mental rotation by subtracting the brain activity associated with scrambled images. Next, we computed two direct contrasts of interest (hands > full-bodies; full-bodies > hands) to individuate the brain regions recruited more strongly during mental rotation of hands with respect to full-bodies, and vice versa. The resulting activations reflected pure mental processing, expected to be weaker than what is triggered by visual stimulation. Thus, with respect to the (visual) functional localizer, for these two main contrasts we used the same FDR correction for multiple comparisons (p < 0.05; t = 4.0) but a lower cluster size threshold (k > 5)^58^. In order to test the stability of the obtained results, in a stability check analysis we repeated the original second-level analysis and included the RTs as covariate in the statistical model. The activation clusters resulting from this stability check analysis were comparable to the ones resulting from the first analysis, both in terms of location and strength (Fig. S1). On this basis, we can exclude that the obtained results are significantly modulated by RTs and are rather stimulus-dependent activations.

To localize and visualize the activated clusters we used the BrainShow software^61^ implemented in Matlab (The MathWorks Inc., Natick, MA, USA). In particular, we projected the group activations onto the cortical surface of the PALS atlas^62^ and superimposed them to a standard template brain. Then, for each cluster, we computed the size (mm^3^) and the percentage of voxels belonging to BAs and anatomical structures^63^.

## Additional Information

**How to cite this article**: Perruchoud, D. *et al*. Differential neural encoding of sensorimotor and visual body representations. *Sci. Rep.* **6**, 37259; doi: 10.1038/srep37259 (2016).

**Publisher's note:** Springer Nature remains neutral with regard to jurisdictional claims in published maps and institutional affiliations.

## Supplementary Material

Supplementary Material

## Figures and Tables

**Table 1:** Activated clusters.

**Region**	**Hemisphere**	**T-value**	**Cluster size (voxels)**	**Peak (MNI)**		
				***x***	***y***	***z***
**Hand** > **Body**						
SMA	L	5.24	28	−4	22	60
	R	4.26	6	8	−2	62
PMC	L	7.01	99	−22	−16	66
	R	5.63	11	32	−18	66
SII	L	6.49	59	−58	−30	34
	L	4.53	11	−40	−30	40
BG	L	5.78	60	−16	10	2
	R	5.76	43	18	12	2
**Body** > **Hand**						
MOG	R	13.84	547	26	−94	12
MOG	L	13.01	210	−16	−102	8
FG	L	4.95	36	−36	−48	−16
EBA	L	4.02	7	−42	−84	−6

Regions more strongly activated during mental rotation of hands with respect to full-bodies were included in the sensorimotor network. The visual network was more active during mental rotation of full-bodies with respect to hands.
