# Supplementary material for: Differential neural encoding of sensorimotor and visual body representations
Source: Sci Rep. 2016 Nov 24;6:37259. doi: 10.1038/srep37259 (PMC5121642; doi:10.1038/srep37259)
Supplement: Supplementary Material [file srep37259-s1.pdf]

SUPPLEMENTARY MATERIAL
for

DIFFERENTIAL NEURAL ENCODING OF SENSORIMOTOR AND VISUAL BODY REPRESENTATIONS

David Perruchoud[1#], Lars Michels[2#], Marco Piccirelli[2], Roger Gassert[3], Silvio Ionta[1,3,*]

1. The Laboratory for Investigative Neurophysiology (The LINE), Department of Radiology and Department of Clinical Neurosciences, University Hospital Center (CHUV) and University of Lausanne (UNIL), Lausanne, Switzerland

2. Institute of Neuroradiology, University Hospital Zurich, Zurich, Switzerland

3. Rehabilitation Engineering Laboratory, Department of Health Sciences and Technology, ETH Zürich, Zurich, Switzerland

* Corresponding Author
# contributed equally

All correspondence should be addressed to:
Silvio Ionta
CHUV, Avenue Pierre Decker 5, 1011 Lausanne, Switzerland
voice: +41213148085
email: ionta.silvio@gmail.com

Methods

Behavioral data - Post-hoc comparisons were carried out using the Bonferroni correction ($p<0.05$). Following earlier analysis pipelines[1], trials whose RTs were shorter than 500ms or longer than 2800ms were excluded from the analysis, with a total of loss of 3.5% of the responses.

fMRI data were corrected for head motion and slice-timing, coregistered with the corresponding anatomical scan, normalized to the MNI template, and smoothed using a 6mm FWHM spatial filter to augment the signal-to-noise ratio[2].

Results

Behavior - The significant 3-way interaction between stimulus, side, and orientation [($F(3,45)=6.24$; $p<0.05$], indicated that the typical mental rotation function was preserved for both right- and left-lateralized hands, as well as for left-lateralized full-bodies. However, despite the typical progressive increase in RTs for right-lateralized full-bodies at 0°, 90°, and 180° (all $p<0.05$), the RTs for images at 270° were not statistically different with respect to 180° ($p>0.1$).

The significant 2-way interaction between stimulus and side ($F(1,15)=5.62$; $p<0.05$) indicated that, with each category (hands, full-bodies), the speed to mentally rotate left- and right-lateralized images was equivalent (all $p>0.1$). Other effects generally confirmed previous findings on the increase of RTs as a function of stimulus orientation [3].

Figure S1

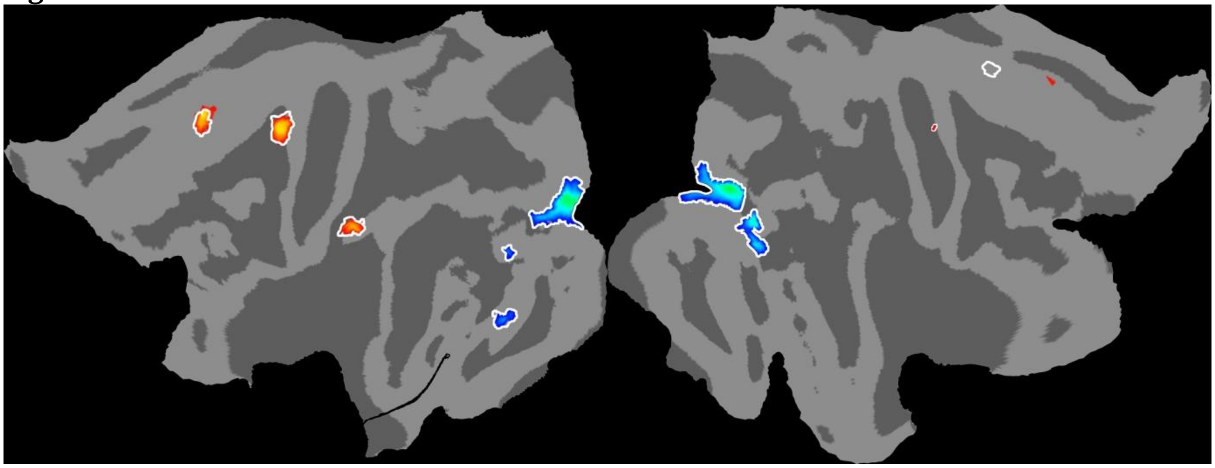

Figure S1. Stability Check. The activation clusters resulting from the stability check analysis performed with RTs as covariate reasonably overlapped (white outline) with the ones resulting from the first second-level analysis (red-to-yellow and blue-to-green).

References

1. Kosslyn, S. M., DiGirolamo, G. J., Thompson, W. L. & Alpert, N. M. Mental rotation of objects versus hands: neural mechanisms revealed by positron emission tomography. *Psychophysiology* **35**, 151-161 (1998).

2. Friston, K. J., Worsley, K. J., Frackowiak, R. S., Mazziotta, J. C. & Evans, A. C. Assessing the significance of focal activations using their spatial extent. *Hum Brain Mapp* **1**, 210-220, (1994).

3. Ionta, S., Fourkas, A. D., Fiorio, M. & Aglioti, S. M. The influence of hands posture on mental rotation of hands and feet. *Exp Brain Res* **183**, 1-7, (2007).

Table S1	LH			RH		
	x	*y*	*z*	*x*	*y*	*z*
2001						
Downing et al.	-51	-72	8	51	-71	1
2002						
Grossman & Blake	-39	-73	11	41	-68	8
2004						
Astafiev et al.	-52	-74	5	-	-	-
Chan et al.	-46	-75	0	46	-71	-3
2005						
Peelen & Downing, c	-43	-74	-7	46	-72	-5
Urgesi et al.	-	-	-	55	-75	-1
Peelen & Downing, b	-46	-76	-8	43	-70	-4
Sakreida et al.	-37	-76	11	41	-73	6
2006						
Arzy et al.	-44	-71	-1	-	-	-
Downing et al., b	-	-	-	43	-72	-4
Spiridon et al.	-64	-83	21	33	-77	2
Downing et al., a	-46	-71	1	46	-69	2
Morris et el.	-42	-83	9	56	-82	9
2007						
David et al.	-50	-76	10	52	-70	5
Downing et al.	-48	-69	3	48	-72	-1
Taylor et al.	-48	-73	-4	50	-67	1
Grezes et al.	-44	-82	4	54	-72	6
Urgesi et al.	-53	-71	4	52	-73	4
2008						
Hodzic et al.	-46	-70	0	48	-64	0
Lamm & Decety	-54	-69	9	54	-67	8
Pichon et al.	-44	-86	0	53	-76	0
2010						
Blanke et al.	-45	-83	-4	-	-	-
Calvo-Merino et al.	-55	-74	0	55	-74	0
2011						
Ionta et al.	-46	-74	2	50	-70	0
2012						
Sinke et al.	-42	-76	5	-	-	-
Tomasino et al.	-42	-76	4	46	-76	-4
2015						
Beck et al.	-47	-72	7	-	-	-
Engelen et al.	-	-	-	47	-74	-3
2016						
Cazzato et al.	-53	-74	0	52	-74	0
Limanowski & Blankenburg	-46	-82	-2	46	-78	0
**Mean (±StDev)**	**-47.1 (±5.6)**	**-75.4 (±4.8)**	**3.3 (±6.3)**	**48.3 (±5.5)**	**-72.3 (±3.9)**	**1.1 (±4.1)**

Table S1. EBA location. Mean and standard deviation of the EBA coordinates over 30 studies. The centroids of the EBA clusters found in the present study (-43, -83, -6 and 41, -81, -1) were within the range of coordinates reported in previous studies.
